# Prevalence and risk factors of extended-spectrum beta-lactamase producing *E. coli* causing urinary tract infections in Iceland during 2012–2021

**DOI:** 10.1007/s10096-024-04882-z

**Published:** 2024-06-27

**Authors:** Anna Margrét Halldórsdóttir, Birgir Hrafnkelsson, Kristjana Einarsdóttir, Karl G. Kristinsson

**Affiliations:** 1 Centre for Health Security and Communicable Disease Control, Directorate of Health, Reykjavík, Iceland; 2 Department of Mathematics, Faculty of Physical Sciences, University of Iceland, Reykjavík, Iceland; 3 Centre of Public Health Sciences, Faculty of Medicine, University of Iceland, Reykjavík, Iceland; 4 Department of Clinical Microbiology, Landspitali, The National University Hospital of Iceland, Reykjavík, Iceland; 5 Faculty of Medicine, University of Iceland, Reykjavík, Iceland

**Keywords:** *Escherichia coli*, Antimicrobial resistance, Antimicrobial agents, Antibiotics, Urinary tract infections, Risk factors, Beta-lactamases

## Abstract

**Purpose:**

To investigate the association of potential risk factors for urinary tract infections (UTI) caused by *E. coli* producing ESBL vs. not producing ESBL in Iceland.

**Methods:**

Observational, case-control study including a cohort of 27,747 patients (22,800 females, 4,947 males; 1207 cases, 26,540 controls) of all ages with UTI caused by *E. coli* in 2012 to 2021 at the clinical microbiology laboratory covering about 2/3 of the Icelandic population. Clinical patient data was obtained from three national databases. Logistic regression was used to calculate odds ratios (ORs) and 95% confidence intervals (CIs) as a measure of association between ESBL and exposure variables.

**Results:**

The proportion of samples with ESBL-producing *E. coli* increased during the study period, from 2.6% in 2012 to 7.6% in 2021 (*p* < 0.001). ESBL-positive strains were detected in 1207 individuals (4.4%), 905 females (4.0%) and 302 males (6.1%). The following risk factors were identified: Male sex, higher age, institution type (hospital, nursing home), hospital-associated UTI, Charlson comorbidity index score ≥ 3, history of cystitis or hospitalization in the past year, and prescriptions for certain antibiotics or proton pump inhibitors (PPIs: OR 1.51) in the past half year. The antibiotic associated with the highest risk was ciprofloxacin (OR 2.45).

**Conclusion:**

The prevalence of UTIs caused by ESBL-producing *E. coli* has been increasing in Iceland. The strongest risk factors for ESBL production were previous antibiotic use, especially ciprofloxacin, and previous PPI use, both considered to be overprescribed. It is important to promote the prudent use of these drugs.

**Supplementary Information:**

The online version contains supplementary material available at 10.1007/s10096-024-04882-z.

## Introduction

β-lactamases are a major factor in the rise of antibiotic resistance in Gram-negative bacteria [1]. The extended-spectrum β-lactamases (ESBL) confer resistance to penicillins, cephalosporins and monobactams [2]. The incidence of infections due to ESBL-producing bacteria has increased rapidly in recent years and is considered a worldwide threat to health care [3, 4].

ESBL-producing *Escherichia coli (E. coli)* is increasingly implicated in community-acquired (CA) infections, especially urinary tract infections (UTIs) [5]. In a recent meta-analysis several risk factors emerged as most relevant for CA-UTI due to ESBL-producing *Enterobacterales*: Prior use of antibiotics, previous hospitalization, and UTI history [3]. Other factors include recent travel to high-prevalence areas (especially Asia or Africa) and food choice [6]. Published studies differ considerably with regards to identified risk factors suggesting that the profile may depend on the study population as well as location [3].

The impact of ESBL-producing *Enterobacterales* on the choice of antimicrobial therapy is reflected in the increased use of carbapenems [7]. Carbapenemase-producing *Enterobacterales* are a growing threat to public health and were defined as Priority 1 (critical) antibiotic-resistant pathogens by the WHO in 2017 [4, 8]. Presumably, many ESBL-related risk factors are also risk factors associated with carbapenemases.

The aim was to investigate the prevalence and risk factors for UTI associated with ESBL-producing *E. coli* in Iceland. The results of this and similar studies may help control the spread of β-lactamases, including carbapenemases.

## Methods

### Ethical considerations

The study was approved by the National Bioethics Committee in Iceland (VSNb2021050039/03.01) and the Research Committee for Health Care Research at Landspitali University Hospital (210,603, ref. 16). This was a non-intervening register-based study and as such did not require informed consent from individual participants.

### Study design and population

The design was an observational case-control study within a cohort of all patients diagnosed with UTI caused by *E. coli* registered in the laboratory information system at the Clinical Microbiology Department at Landspitali University Hospital (CM-LUH) in Reykjavik in the period of January 1st 2012 to June 30th 2021, irrespective of age. Patients whose first positive urine culture was recorded after 48 h of hospitalization were defined as having hospital-associated UTIs (HA-UTI). Other patients were regarded as CA-UTI. Inclusion in the study was therefore based on positive *E. coli* urine culture results at CM-LUH rather than on clinical ICD-10 diagnostic codes for UTIs. Furthermore, information on clinical presentation was not available in the laboratory information system and therefore not collected in this study.

Patients were assigned as *cases* if they had a positive urine culture with ESBL-producing *E. coli* at least once in the study period, while the remaining patients with non-ESBL-producing *E. coli* isolates were assigned as *controls*. Only a single ESBL-producing bacterial isolate per patient was included, i.e. the isolate with the oldest sample date in the study database if the patient had multiple ESBL-positive samples. For patients in the control group with multiple positive urine cultures, only the first sample was included.

### Data extraction

Data on both cases and controls were extracted from the laboratory information system (LIS) of CM-LUH. The following data were retrieved through the LIS; personal identification number (PIN), demographic data (age and sex), postal code, results of urine culture and antibiotic susceptibility tests (including presence of ESBL) and ordering department/institution. Antimicrobial susceptibility testing and ESBL detection and confirmation was performed according to the EUCAST methods and criteria (www.eucast.org).

Information on potential risk factors for ESBL-producing *E. coli* associated UTIs were extracted from the following nation-wide centralised registries maintained at, or under the auspices of, the Directorate of Health (DoH): the Hospital Discharge Register, the Register of Primary Health Care Contacts, and the Prescription Medicines Register. Data from CM-LUH was merged with data from the registries mentioned above using encrypted personal identification numbers, unique numbers assigned to each resident at birth or immigration. The data merging was implemented at the DoH in Iceland. Subsequent data handling was anonymized.

### Exposure and covariate variables

Information on previous exposure to pharmaceuticals and previous medical history was collected from the following registers. The Prescription Medicines Register (up to six months before the index UTI): (1) Antibiotic use (all prescriptions within the entire ATC J01 category to fifth level); (2) Proton pump inhibitors (ATC A02BC); (3) Corticosteroids (ATC H02AB), antineoplastic agents (ATC L01), and Immunosuppressants (ATC L04). The Hospital Discharge Register/Register of Primary Health Care Contacts: (1) Diagnoses included in the Charlson co-morbidity index (CCI) score, to adjust for the presence of underlying chronic conditions; (2) Previous UTIs for the past one year before the index UTI (N30 Cystitis); (3) Any type of surgery for the past one year before the index UTI; (4) Any hospitalization for the past one year before the index UTI.

### Statistical analysis

Logistic regression was used to estimate crude and adjusted ORs and 95% CIs. Exposure factors were compared between cases (ESBL-positive) and controls (ESBL-negative). Potential covariates were age, sex, and the Charlson co-morbidity index (CCI) score. The CCI score was classified as three comorbidity levels: low (score of 0), medium (score of 1–2) and high (score ≥ 3) [9, 10]. Analysis of risk factors was performed for the overall group first and then separately for males and females and various age groups (< 1, 1–18, 18–60, ≥ 60 years). Each coefficient in the multivariate logistic regression model was considered statistically significant if its two-tailed p value was < 0.05. Multivariate logistic regression models were assessed by the Hosmer and Lemeshow Goodness of Fit Statistic. Statistical analyses were performed using Microsoft Excel spreadsheets and R studio version 4.1.2 (2021-11-01), an integrated development environment for the statistical software R.

## Results

### The study group

**Fig. 1:**
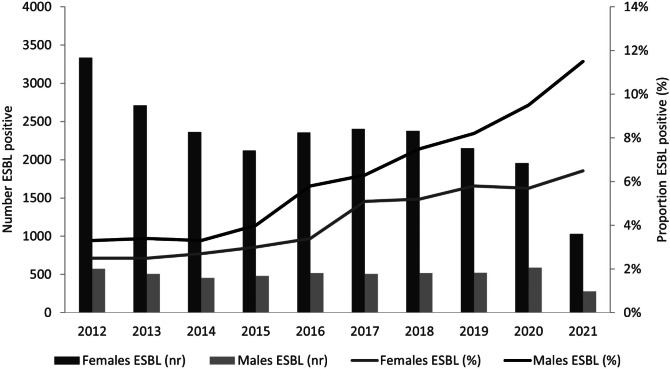
The proportion of ESBL positive E. coli positive urine samples and the total number of urine samples, by sex and year. For the calendar year 2021 only the first six months were included in the study. For the ESBL-positive group only the first ESBL-positive urine sample for each individual in the study period was included in the study. For the ESBL-negative group only the first urine sample for each individual in the study period was included in the study

A total of 27,747 individuals, 22,800 females and 4,947 males, were identified with UTI caused *by E. coli*. The most common urine sample types were mid-stream urine for 13.159 (47.4%) cases, first-stream urine for 2.898 (10.4%), urinary catheter urine for 1.600 (5.8%) and clean catch urine for 1.314 (4,7%) cases, while no sample type was specified for 5.904 (21.3%) individuals and miscellaneous types constituted the remaining specimens. The median age was 56.1 years (range 0-106 years), 65.8 years for males and 52.8 years for females (supplemental Fig. 1). The proportion of samples with ESBL-producing *E. coli* increased over the study period, from 2.6% in 2012 to 7.6% in 2021 (*p* < 0.001, Fig. 1). Overall, ESBL-producing *E. coli* isolates were detected in 1207 individuals (4.4%), 905 females (4.0%) and 302 males (6.1%). ESBL was most frequent in individuals ≥ 60 years (5.5%) and least frequent in children 1–18 years (2.4%). The highest ESBL rate was observed in nursing home samples (6.3%) and the lowest in primary emergency care samples (2.8%) (Table 1).

**Table 1:** Baseline characteristics of study participants with UTI (*n* = 27,747) by ESBL status in Iceland during 2012–2021

Variables	Number (*n*)	ESBL positive (%)	ESBL status *n* = 27,747		
			Positive, *n* (%) *n* = 1,207	Negative, *n* (%) *n* = 26,540	*p*-value
**UTI category**					
CA-UTI	25,775	4.1%	1067 (88.4)	24,708 (93.1)	< 0.001
HA-UTI	1972	7.1%	140 (11.6)	1832 (6.9)	
**Institution**					
Landspitali	13.304	4.9%	652 (54.0)	12,652 (47.7)	< 0.001
Primary care	9794	3.8%	372 (30.8)	9422 (35.5)	
Private clinic	2402	3.5%	85 (7.0)	2317 (8.7)	
Primary emergency care	999	2.8%	28 (2.3)	971 (3.7)	
Nursing home	941	6.3%	59 (4.9)	882 (3.3)	
Other	306	3.6%	11 (0.9)	295 (1.1)	
**Antibiotics (ATC 5th level)**					
Pivmecillinam J01CA08	6.851	4.6%	316 (26.2)	6535 (24.6)	0.233
Amoxicillin/β-lactam. inh. J01CR02	2.846	5.4%	153 (12.7)	2693 (10.1)	0.005
Nitrofurantoin J01XE01	2.249	5.7%	128 (10.6)	2121 (8.0)	0.001
Amoxicillin J01CA04	2.271	5.7%	129 (10.7)	2142 (8.1)	0.001
Ciprofloxacin J01MA02	2.373	9.2%	219 (18.1)	2154 (8.1)	< 0.001
Sulfameth.-trimetoprim J01EE01	1.617	5.8%	94 (7.8)	1523 (5.7)	0.004
Trimethoprim J01EA01	1.436	5.9%	85 (7.0)	1351 (5.1)	0.003
Cefalexin J01DB01	1.305	5.8%	76 (6.3)	1229 (4.6)	0.009
Doxycyclin J01AA02	1.154	5.3%	61 (5.1)	1093 (4.1)	0.129
Azithromycin J01FA10	1.081	6.3%	68 (5.6)	1013 (3.8)	0.002
**Other medications**					
Proton pump inhibitors (PPIs)	6492	6.8%	441 (36.5)	6051 (22.8)	< 0.001
Corticosteroids	2884	6.4%	186 (15.4)	2698 (10.2)	< 0.001
Antineoplastic / immunosuppressant	891	7.5%	67 (5.6)	824 (3.1)	< 0.001
**Medical history**					
Cystitis	7539	5.3%	399 (33.1)	7140 (26.9)	< 0.001
Hospitalization	7498	6.3%	471 (39.0)	7027 (26.5)	< 0.001
Surgery	1760	6.0%	105 (8.7)	1655 (6.2)	0.001
**Charlson co-morbidity index**					
None (no data)	6022	3.1%	187 (15.5)	5835 (22.0)	< 0.001
Zero (0)	13,286	3.8%	511 (42.3)	12,775 (48.1)	
Low (1–2)	7350	5.4%	400 (33.1)	6950 (26.2)	
High (3+)	1089	10.0%	109 (9.0)	980 (3.7)	

### Previous Pharmaceutical prescriptions and Medical History

Some antibiotics were more frequently prescribed for females (pivmecillinam, nitrofurantoin, trimethoprim) and others (ciprofloxacin, amoxicillin/β-lactamase inhibitor, doxycycline) for males (supplemental Fig. 3a). Antibiotic prescriptions also differed between age groups (supplemental Fig. 2b).

A higher proportion of ESBL positive than ESBL negative study participants had received antibiotics, proton pump inhibitors (PPIs), oral corticosteroids, and antineoplastic/immunosuppressant agents (Table 1). More ESBL positive than ESBL negative individuals had a history of cystitis, hospitalisation, or surgery (Table 1). The mean CCI score was higher for ESBL positive than ESBL negative individuals (0.93 vs. 0.59, *p* < 0.001) and a higher proportion of ESBL positive subjects had a score of ≥ 3 (Table 1).

### Univariate and Multivariate Risk factor analysis

The risk of having ESBL-producing *E. coli* in urine was analysed separately for sex and age subgroups. Male sex and higher age were each associated with increased risk for ESBL in both univariate and multivariate logistic models (Table 2). The Hosmer-Lemeshow goodness of fit test for the multivariate model indicated an acceptable fit to the data (X-squared = 30.9, p-value = 0.3).

**Table 2:** ESBL risk according to sex and age-group for study participants with UTI (*n* = 27,747) in Iceland during 2012–2021

Group	Number	Crude OR	95% CI	*p*-value	Adjusted OR ^a)^	95% CI	*p*-value
**Sex**							
Males	4,947	1.57	1.38–1.80	< 0.001	1.36	1.17–1.57	< 0.001
Females	22,800	1.0	-	-	1.0	-	-
**Age (years)**							
< 1	976	1.32	0.85–2.03	0.21	1.26	0.80–1.96	0.32
1–18	3017	1.0	-	-	1.0	-	-
18–60	11,158	1.62	1.25–2.09	< 0.001	1.54	1.17–2.03	< 0.001
≥ 60	12,596	2.39	1.87–3.07	< 0.001	1.66	1.25–2.20	< 0.001

*a) Adjusted for institution type, CCI score, medical history, UTI category, antibiotics, other medications (see* Table 1*)*

The association of selected factors with the risk for ESBL-producing vs. non-ESBL-producing *E. coli* was analysed for the whole study group (Table 3). The Hosmer-Lemeshow goodness of fit test for the multivariate model indicated an acceptable fit to the data (X-squared = 15.7, p-value = 0.97). In this multivariate analysis the following factors were independent risk factors of ESBL producing isolates; type of institution (primary care, hospital, or nursing home), a CCI score ≥ 3, history of cystitis or hospitalization in the past one year, HA-UTI, prescriptions for all antibiotics analysed except amoxicillin with β-lactamase inhibitors, doxycycline and azithromycin, and prescriptions for PPIs (Table 3).

**Table 3:** Risk factors for ESBL in study participants with UTI (*n* = 27,747) in Iceland during 2012–2021

Group	Crude OR	95% CI	*p*-value	Adjusted OR	95% CI	*p*-value
Institution						
Primary emergency clinic	1	-	-	1	-	-
Private outpatient clinic	1.27	0.82–1.96	0.28	1.39	0.90–2.16	0.14
Other	1.29	0.64–2.63	0.48	1.25	0.61–2.58	0.54
Primary care	1.37	0.93–2.02	0.11	1.50	1.01–2.22	0.04
Hospital	1.79	1.22–2.62	< 0.001	1.69	1.14–2.52	0.01
Nursing home	2.32	1.47–3.67	< 0.001	2.77	1.72–4.46	< 0.001
CCI score						
0	1	-	-	1	-	-
1–2	1.44	1.26–1.65	< 0.001	1.13	0.97–1.32	0.12
≥ 3	2.78	2.24–3.45	< 0.001	1.86	1.45–2.39	< 0.001
No data	0.80	0.68–0.95	0.01	1.13	0.93–1.37	0.23
Medical history						
Cystitis	1.34	1.19–1.52	< 0.001	1.31	1.12–1.52	< 0.001
Hospitalization	1.78	1.58–2.00	< 0.001	1.38	1.16–1.63	< 0.001
Surgery	1.43	1.17–1.76	< 0.001	1.02	0.81–1.28	0.85
UTI category						
HA-UTI	1.77	1.47–2.12	< 0.001	1.32	1.07–1.63	0.01
Antibiotics						
All J01	1.39	1.23–1.58	< 0.001	-	-	-
Pivmecillinam J01CA08	1.09	0.95–1.24	0.22	1.20	1.03–1.39	0.02
Amoxicillin/β-lactamase inhibitor J01CR02	1.29	1.08–1.53	< 0.001	1.06	0.88–1.27	0.56
Nitrofurantoin J01XE01	1.37	1.13–1.65	< 0.001	1.22	1.00–1.49	0.05
Amoxicillin J01CA04	1.36	1.13–1.64	< 0.001	1.26	1.04–1.53	0.02
Ciprofloxacin J01MA02	2.51	2.15–2.92	< 0.001	2.45	2.09–2.88	< 0.001
Sulfamethoxazol / trimethoprim J01EE01	1.39	1.12–1.72	< 0.001	1.51	1.21–1.89	< 0.001
Trimethoprim J01EA01	1.41	1.13–1.77	< 0.001	1.35	1.07–1.72	0.01
Cefalexin J01DB01	1.38	1.09–1.76	0.01	1.61	1.26–2.06	< 0.001
Doxycyclin J01AA02	1.24	0.95–1.61	0.11	1.05	0.80–1.38	0.72
Azithromycin J01FA10	1.50	1.17–1.94	< 0.001	1.22	0.94–1.58	0.14
Other medications						
PPIs	1.95	1.73–2.20	< 0.001	1.51	1.32–1.73	< 0.001
Corticosteroids	1.61	1.37–1.89	< 0.001	0.99	0.82–1.18	0.87
Antineoplastic / immunosuppressants	1.83	1.42–2.37	< 0.001	1.25	0.95–1.64	0.11

The antibiotics associated with the largest OR in the multivariate analysis were ciprofloxacin (OR 2.45), cefalexin (OR 1.61), and sulfamethoxazole-trimethoprim (OR 1.51). Prescriptions for PPIs were a strong risk factor (OR 1.51), as were nursing homes (OR 2.77) and a CCI score ≥ 3 (OR 1.86) (Table 3).

### Multivariate analysis of ESBL risk factors stratified by sex and age

**Fig. 2:**
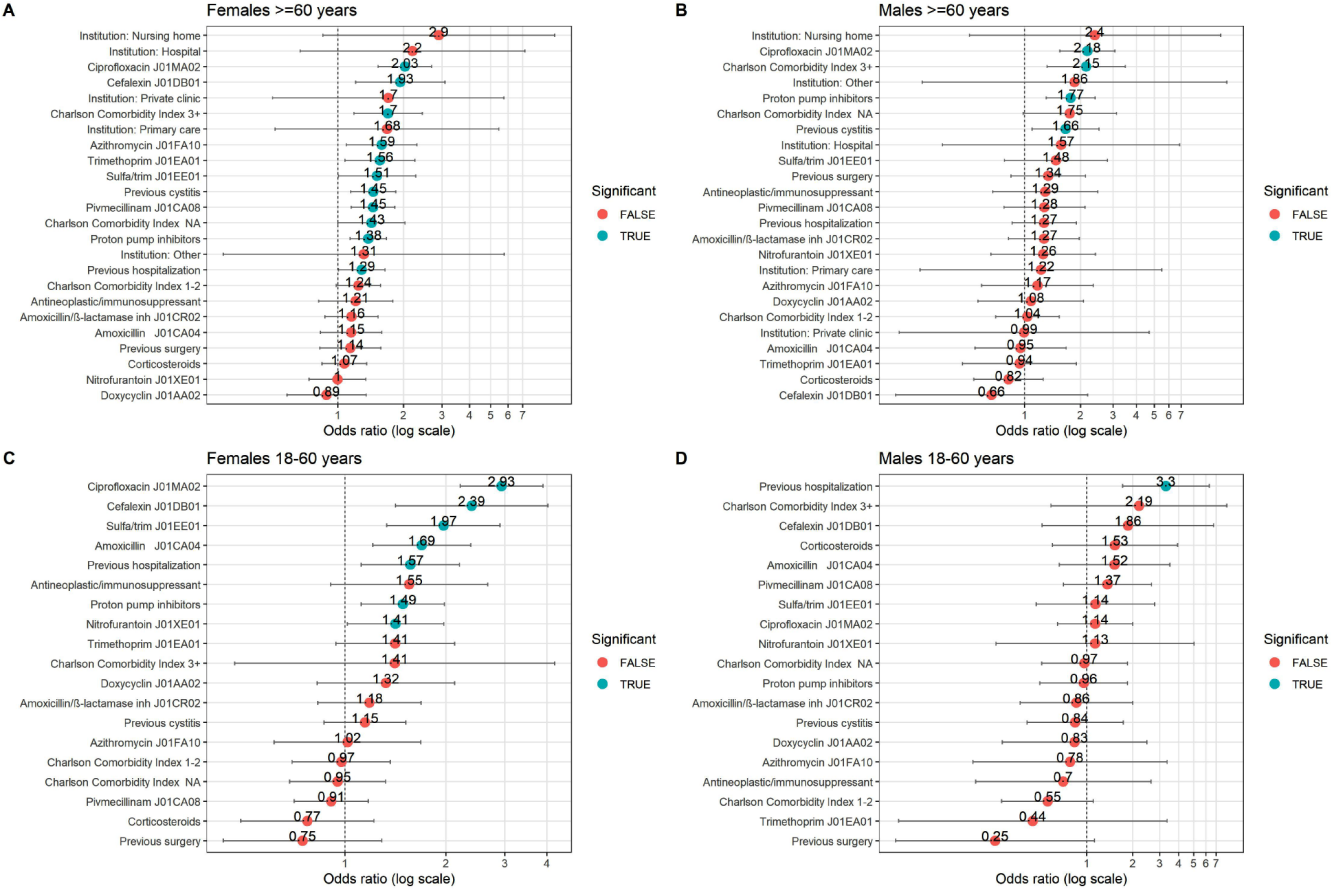
Adjusted OR for selected factors stratified by sex for the two oldest age groups, ≥ 60 years and 18–60 years. Panels **(a)** Females ≥ 60 years (*n* = 9604), **(b)** Males ≥ 60 years (*n* = 2992), **(c)** Females 18–60 years (*n* = 9927), **(d)** Males 18–60 years (*n* = 1231). Significant indicates a p-value < 0.05

Multivariate analysis was performed separately for eight subgroups (eight models) based on sex (males, females) and age groups (< 1, 1–18, 18–60, ≥ 60 years) (Fig. 2, supplemental Fig. 3). However, not all factors were included in all models as some subgroups were small, limiting the power of the analysis. Overall, the Hosmer-Lemeshow goodness of fit test for these models indicated an acceptable fit to the data (p-values > 0.05). The following risk factors emerged:

- Females ≥ 60 years; previous cystitis (OR 1.45), hospitalisation (OR 1.29), CCI score ≥ 3 (OR 1.70), PPI prescriptions (OR 1.38), and various antibiotics (highest OR: ciprofloxacin 2.03).
- Males ≥ 60 years; previous cystitis (OR 1.66), CCI score ≥ 3 (OR 2.15), PPI prescriptions (OR 1.77), and ciprofloxacin antibiotics (OR 2.18).
- Females 18–60 years; previous hospitalisation (OR 1.57), PPIs (OR 1.49) and various antibiotics including ciprofloxacin (OR 2.93).
- Males 18–60 years; only previous hospitalization (OR 3.3).
- Females 1–18 years; only cefalexin (OR 1.99).
- Males 1–18 years; none, but the group was small (*n* = 257).
- Females < 1 years: only cefalexin (OR 3.91).
- Males < 1 years: only PPIs prescription (OR 4.56).

Finally, the multivariate analysis for subgroups based on sex/age was repeated using alternative age cut-offs of 15 and 45 years instead of 18 and 60 years (supplemental Figs. 1 and 4). Additional risk factors that emerged included prescriptions for antineoplastic or immunosuppressant agents (OR 2.93) for females 15–45 years, and nitrofurantoin (OR 3.65) prescriptions for females 1–15 years.

## Discussion

The prevalence of ESBL-producing *E. coli* increased in Iceland during the study period. Male sex and higher age were independent risk factors in multivariate analysis. The following risk factors were identified, excluding the effect of sex or age: Institution type, HA-UTI, CCI score ≥ 3, a history of cystitis or hospitalization in the past year, and antibiotic or PPI prescriptions in the past half year.

Overall, 27,747 individuals were included in the study, corresponding to about 11% of the population in the Reykjavik metropolitan area. The number of females was more than four-fold higher than of males, reflecting the higher incidence of UTI in women [11]. The increasing prevalence of ESBL mirrors the worldwide trend since the early 2000s [3]. The overall prevalence of ESBL in our cohort (4,4%) was similar to two French studies of CA-UTIs from 2013 to 2015 (3.3% and 4.2%), but considerably lower than in a 2022 report from Qatar (25.2%) or a 2017 report from Peru (41%) [12–16]. In a Swedish nation-wide study on community carriage of ESBL-producing *E. coli* a carriage rate of 4.7% was identified, and 8.6% in a study from the Netherlands [17, 18].

Most of the identified risk factors in our study have been associated with ESBL-producing *Enterobacterales* in prior studies [13, 19, 20]. In Larramendy´s systematic review the most relevant risk factors were prior use of antibiotics (OR 2.2–21.4), hospitalisation (OR 1.7–3.9), and UTI history (OR 1.3–3.8) [3]. In contrast to a prior report, surgery was only significant in univariate but not multivariate analysis in our study [14]. Immunosuppressive therapy has been reported as a risk factor for ESBL and this association was present in females 15–45 years old in our analysis (supplemental Fig. 4) [20]. In contrast, no association between ESBL and treatment with corticosteroids was identified in our cohort [14].

Previous antibiotic use has consistently been identified as a risk factor for ESBL carriage and UTIs caused by ESBL-producing *Enterobacterales* [3]. The most frequently implicated classes of antibiotics are the beta-lactam antibiotics and fluoroquinolones, although other compounds have also been implicated [3]. The single antibiotic most strongly associated with ESBL in our study was the fluoroquinolone ciprofloxacin, thus confirming previous studies [6, 21]. Ciprofloxacin is currently the only fluoroquinolone registered on the market in Iceland and other fluoroquinolones were not used to any notable degree during the study period. Beta-lactam antibiotics identified as risk factors in our study included the penicillin compounds pivmecillinam and amoxicillin and the first-generation cephalosporin cefalexin.

Pivmecillinam, an oral antimicrobial agent effective against ESBL producing organisms, has been widely used for uncomplicated UTIs, especially in the Nordic countries [22]. In contrast to our findings, Søraas et al. reported that exposure to mecillinam was not associated with ESBL-positive CA-UTI, but the study was small [6]. Richelsen et al. reported that pivmecillinam was a risk factor for community-onset ESBL-positive *K. pneumoniae* bacteraemia [21]. Previous studies have frequently treated penicillins as a single class rather than specific compounds [23, 24]. Our finding that pivmecillinam exposure is a risk factor needs to be confirmed in other studies.

The strong relationship between ESBL and PPIs observed, overall and in several subgroups (adjusted OR 1,5-4.6), was intriguing. Prior studies have reported an increased risk of rectal carriage with ESBL-producing *Enterobacterales* among patients using PPIs and/or other acid suppressants [18, 25, 26]. In a recent case-control study the recent use of PPIs was associated with a 1.5-fold higher risk of acquiring ESBL- or carbapenemase-producing *Enterobacterales* [27]. In some studies, PPIs have been associated with an increased risk of various infections such as community-acquired pneumonia and gastrointestinal infections [28]. The differential impact of PPIs on infections with ESBL-producing compared to non-ESBL-producing organisms is less clear. Few studies have analysed the impact of PPI use on UTIs caused by ESBL-producing *E. coli*. A Danish case-control study did report PPI intake as a risk factor for ESBL *E. coli* UTI compared to non-ESBL *E. coli* UTI, but only in a logistic model adjusted for age, sex and CCI and not in a larger model adjusted for other diseases, antibiotic use and hospitalisation [20]. In contrast, in our study PPIs remained a strong risk factor in multivariate models including multiple variables and in different subgroups. PPIs are probably overprescribed [29]. Potential adverse events associated with long-term use of PPIs include *Clostridium difficile* infection and bacterial gastroenteritis [30]. Notably, increased risk of infections due to ESBL-producing bacteria is not frequently mentioned in reports on side-effects of PPI therapy [30].

A strength of this study is the large and well-defined cohort, without any selection or exclusion of controls, thus minimizing biases and increasing statistical power. Multivariate analysis using logistic regression was used to identify risk factors and to simultaneously adjust for the presence of possible confounding factors. Other strengths include access to data from several national databases and the collection of multiple variables related to previous medical history, including cystitis and surgery. One strength is the analysis of specific antibiotic substances (ATC fifth level) rather than broader categories (ATC third level). Data were collected on all prescriptions for antibiotics within the ATC J01 category and the study should therefore reflect the entire spectrum of antibiotics used clinically in Iceland during the study period.

Potential limitations of this study include its observational and case-control design. In addition, the number of male children included was too small to allow robust statistical analysis. No data on dietary factors or previous travel were available in the databases used [6]. Information on the clinical presentation of the UTIs was not available and therefore patients with asymptomatic bacteriuria may have been included in the study group. Similarly, cystitis and pyelonephritis could not be separated in this study. Additional data not readily accessible through our registers, were the use of in-hospital antibiotics and urinary catheter use. Furthermore, we restricted our analysis to UTIs associated with *E. coli* but excluded other pathogens such as *K. pneumoniae*, as these may have different epidemiology.

In conclusion, the strongest risk factors for ESBL producing isolates in patients with UTI were previous use of antibiotics, especially beta-lactams and fluoroquinolones, and previous use of PPIs. Both antibiotics and PPIs are believed to be overprescribed and not always used for appropriate indications. Therefore, it is important to promote the prudent use of these drugs, by educating physicians and the public about potential side-effects and long-term consequences for global public health.

### Electronic supplementary material

Below is the link to the electronic supplementary material.

Supplementary Material 1

## Data Availability

The data that support the findings of this study are not openly available due to reasons of sensitivity and are available from the corresponding author upon reasonable request. Data are located in controlled access data storage at the Directorate of Health, Iceland.
