# Supplementary material for: Prevalence and risk factors of extended-spectrum beta-lactamase producing E. coli causing urinary tract infections in Iceland during 2012–2021
Source: Eur J Clin Microbiol Infect Dis. 2024 Jun 27;43(9):1689–97. doi: 10.1007/s10096-024-04882-z (PMC11349795; doi:10.1007/s10096-024-04882-z)

**Supplemental information**

Supplemental figure 1.

Density plot presenting the age distribution of study participants by sex and ESBL status. Dashed vertical lines indicate ages of 1, 15 and 45 years.


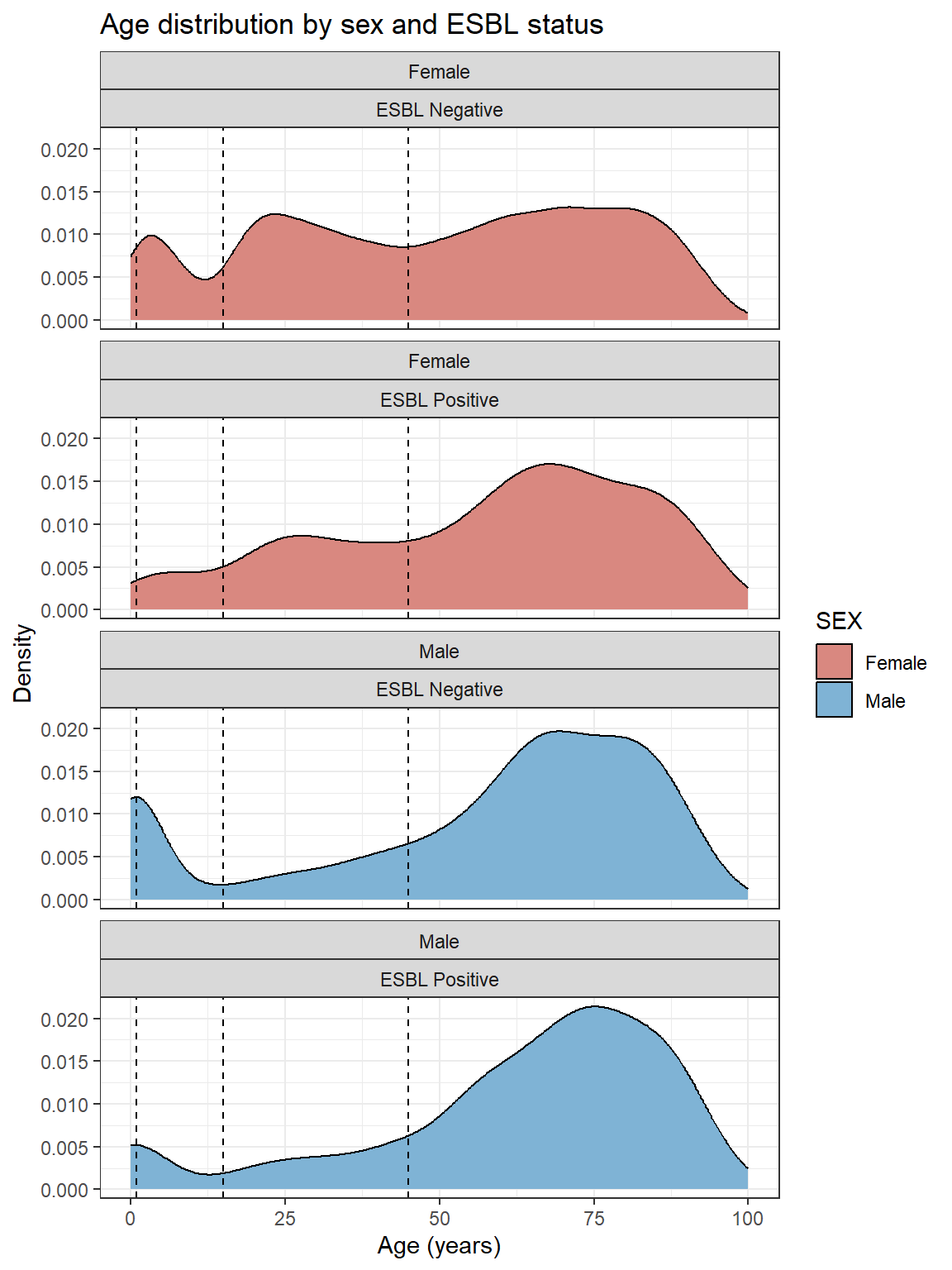


Supplemental figure 2.

1. Antibiotic prescriptions (ATC fifth level) in the study group by sex. The top 16 prescribed antibiotics, sorted by use, are presented in the graph. The number next to each bar represents the percentage of individuals who received prescription for that antibiotic.


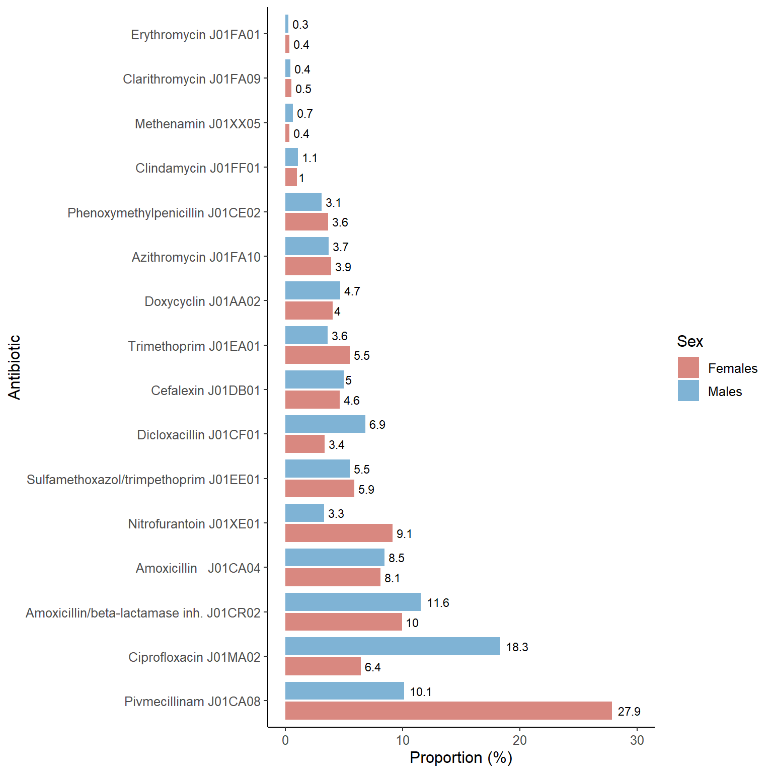


1. Antibiotic prescriptions by antibiotic category (ATC third level), age group and sex.


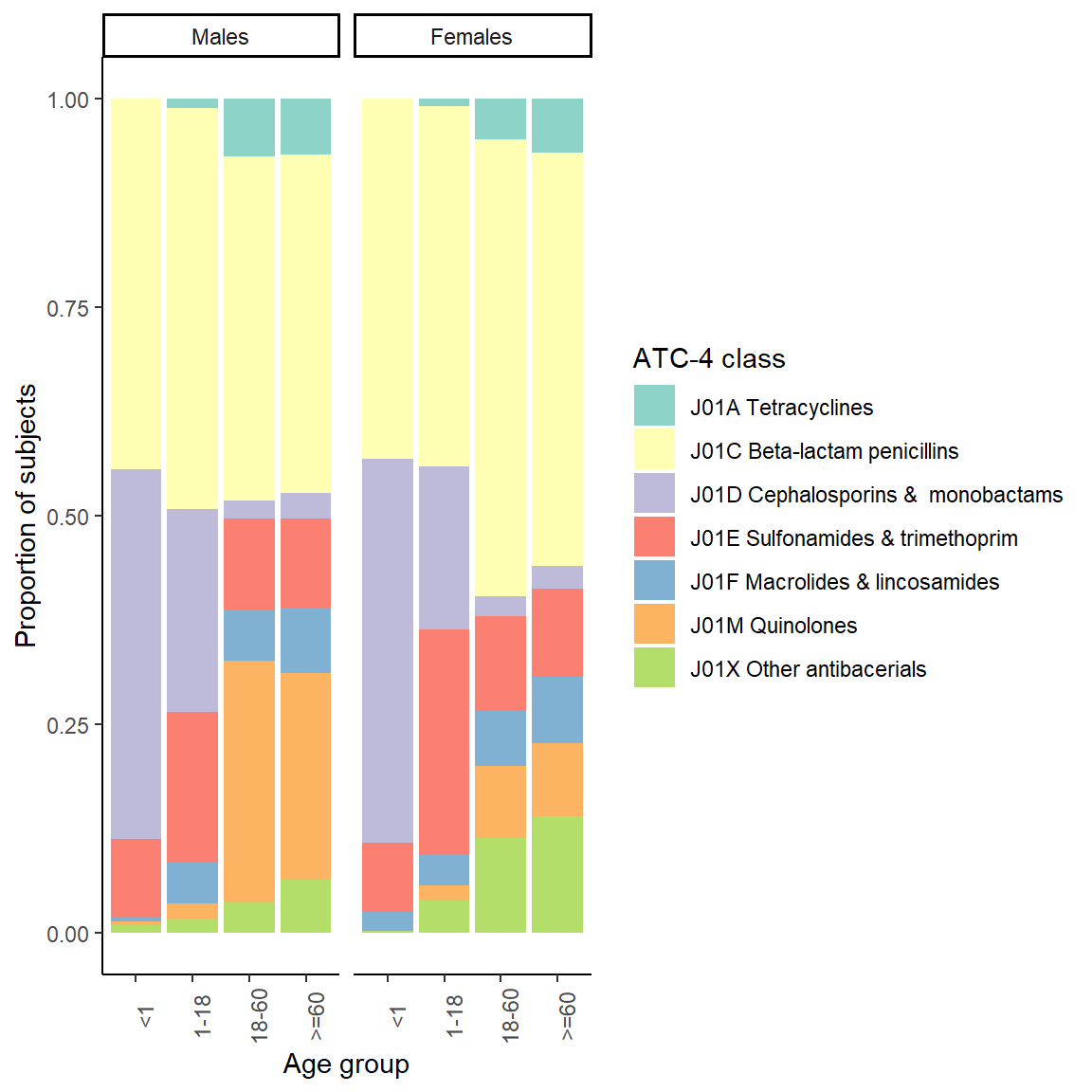


**Supplemental figure 3.**

Adjusted OR for selected risk factors for age groups 1-18 years and <1 years by sex (panels a-d).

1. **Females 1-18 years (n=2760)**


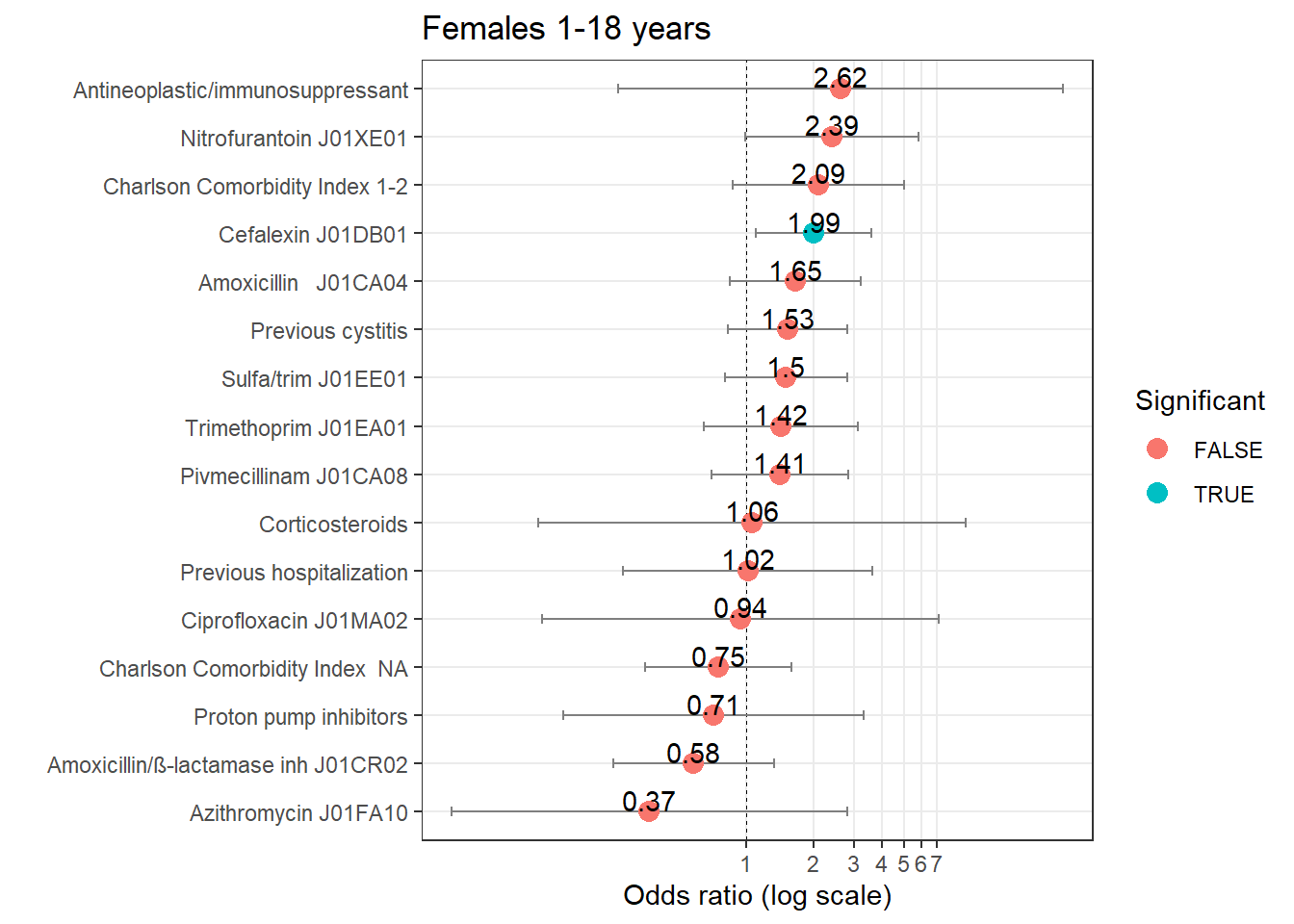


1. **Males 1-18 years (n=257)**


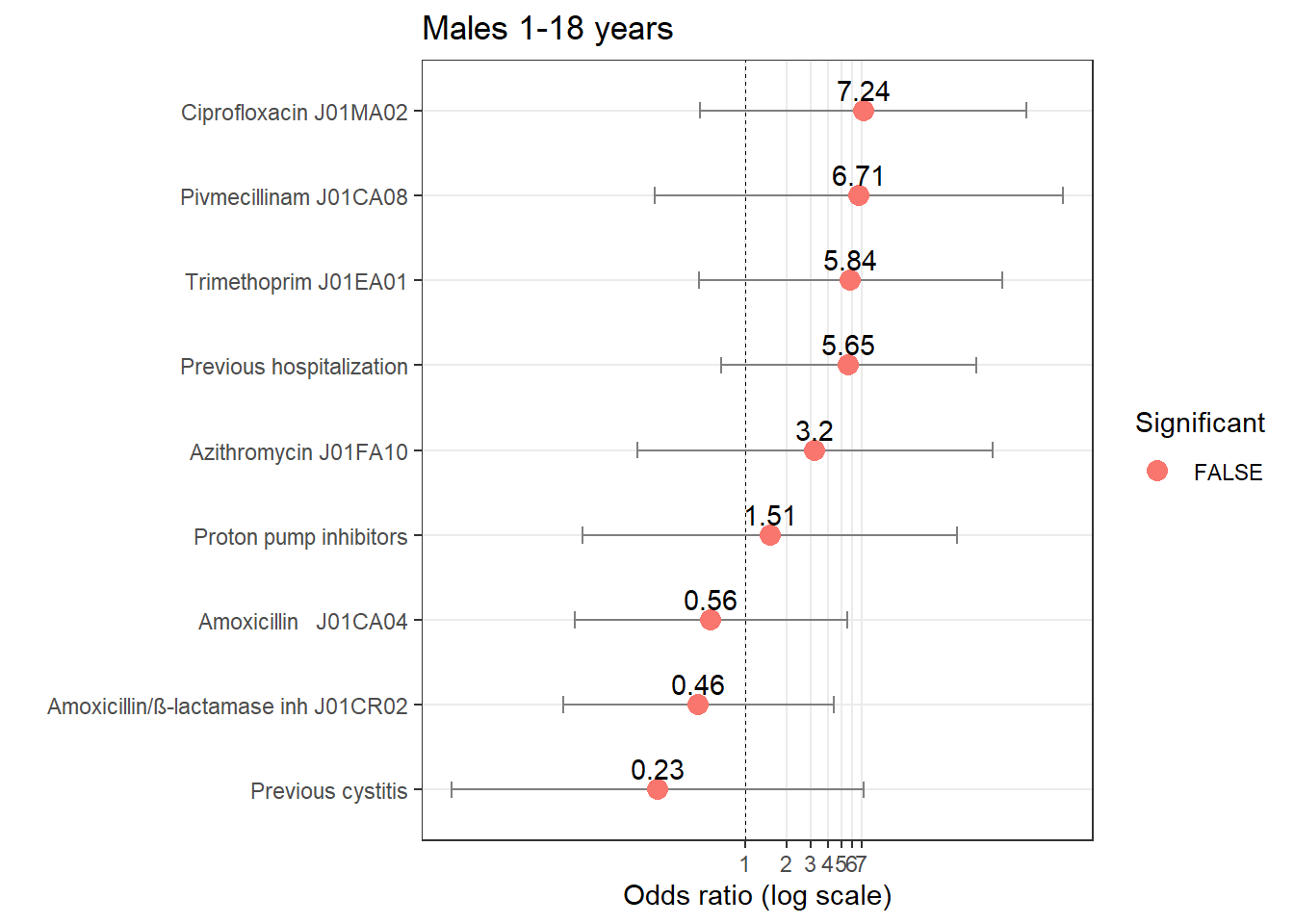


1. **Females <1 years (n=509)**


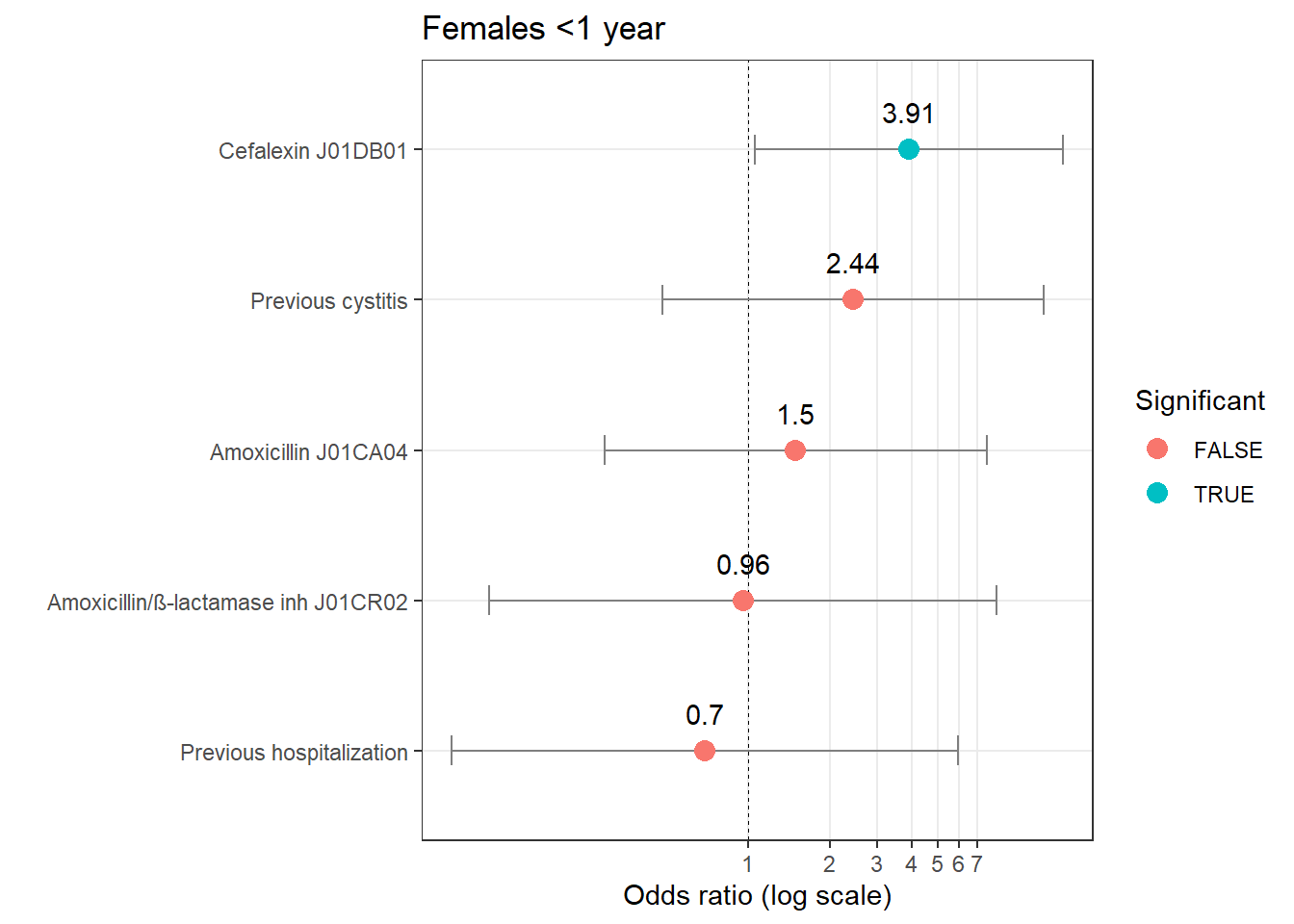


1. **Males <1 years (n=467)**


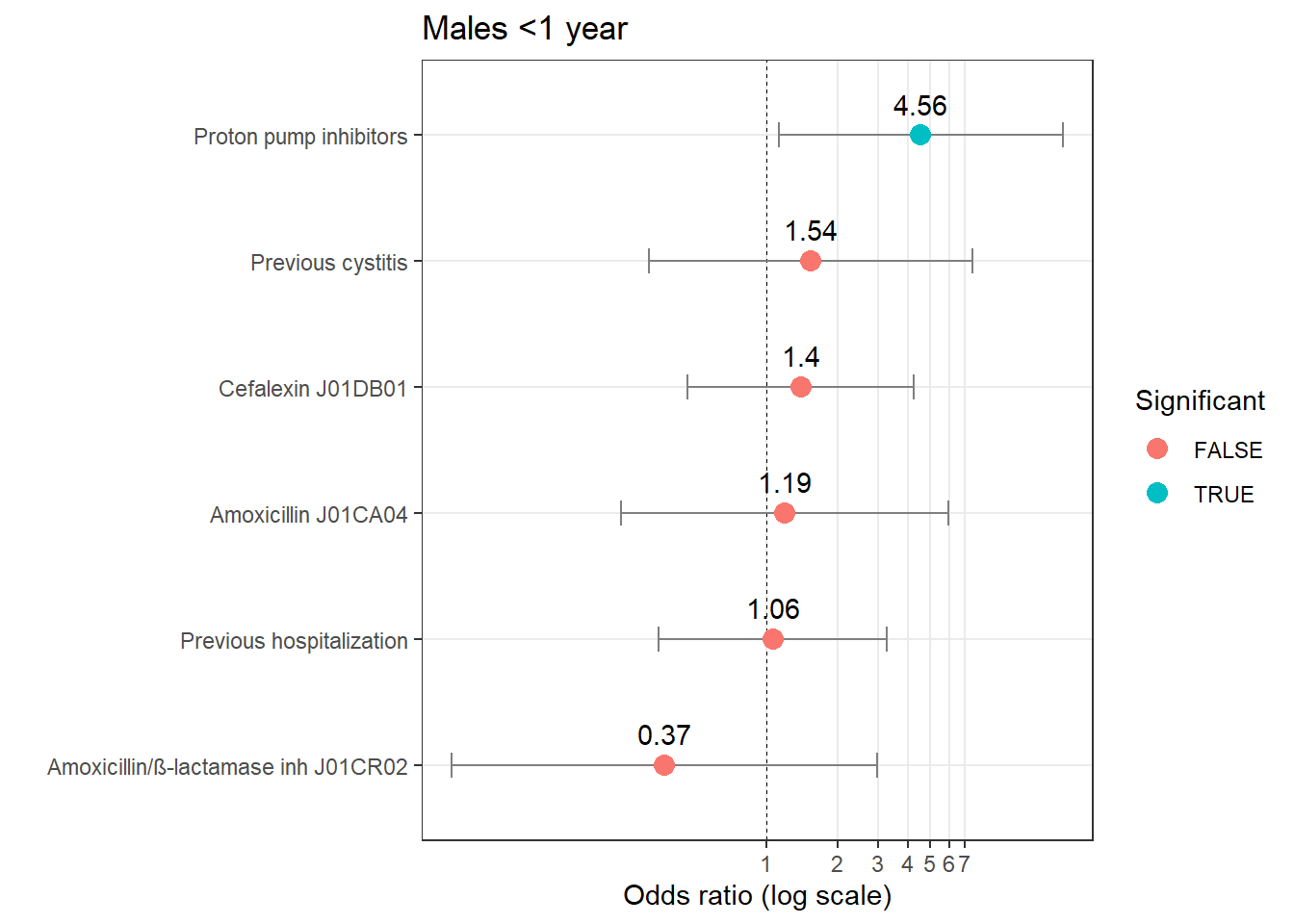


**Supplemental figure 4.**

Adjusted OR for selected risk factors for females using alternative age (15 and 45 years) cutoffs (panels a-f).

1. **Females ≥45 years (n=** **13,012)**


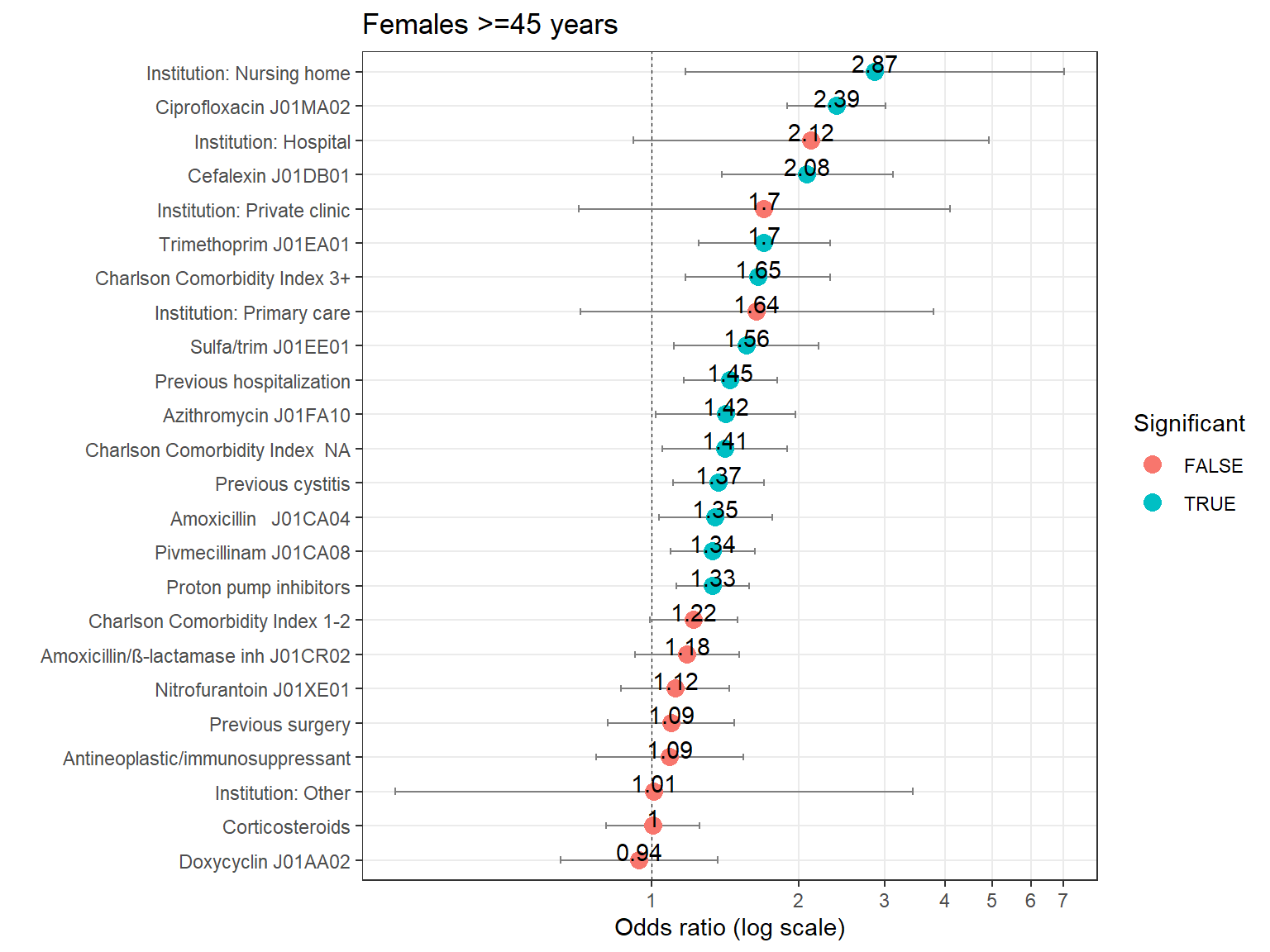


1. **Males ≥45 years (n=3685)**


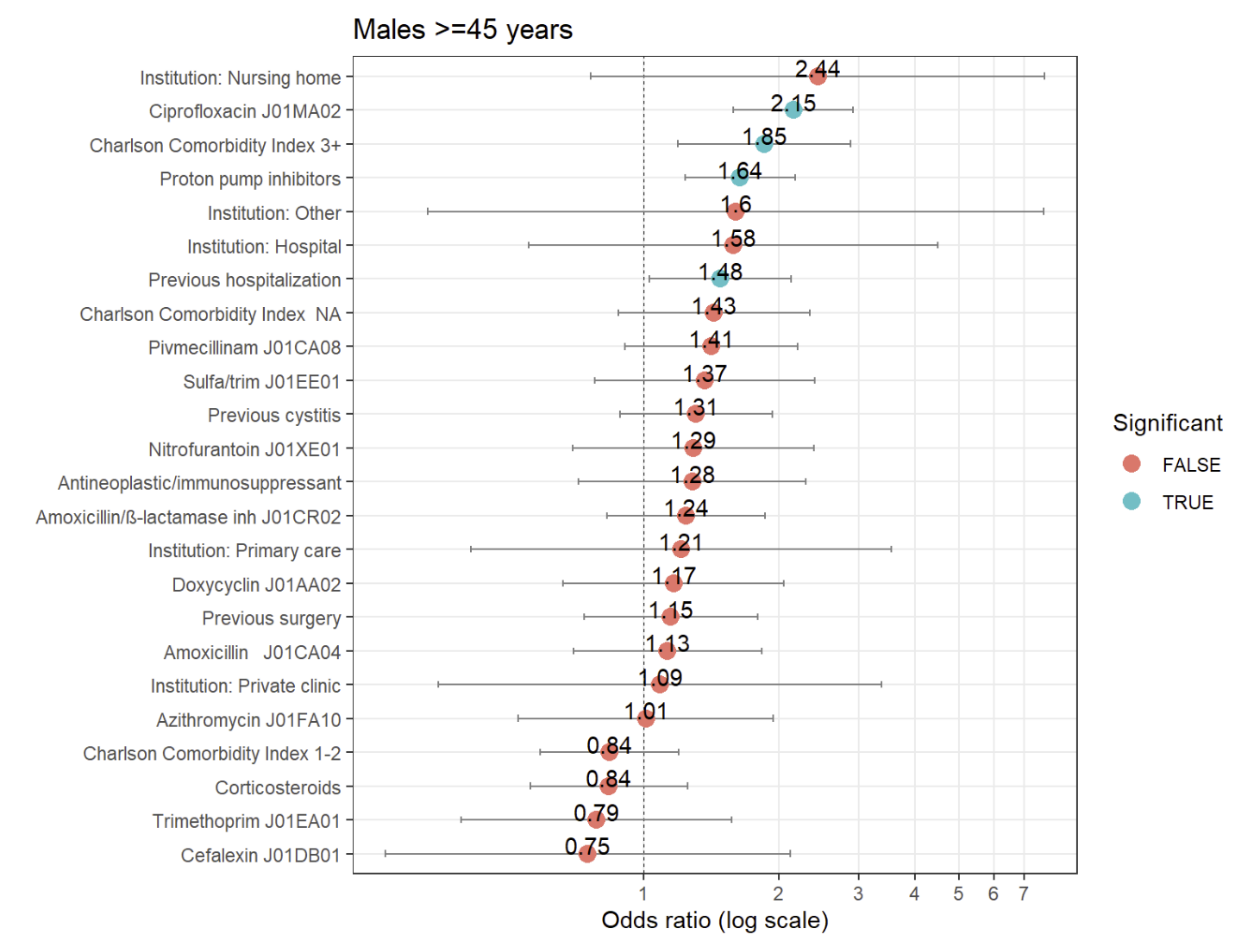


1. **Females 15-45 years (n=7077)**


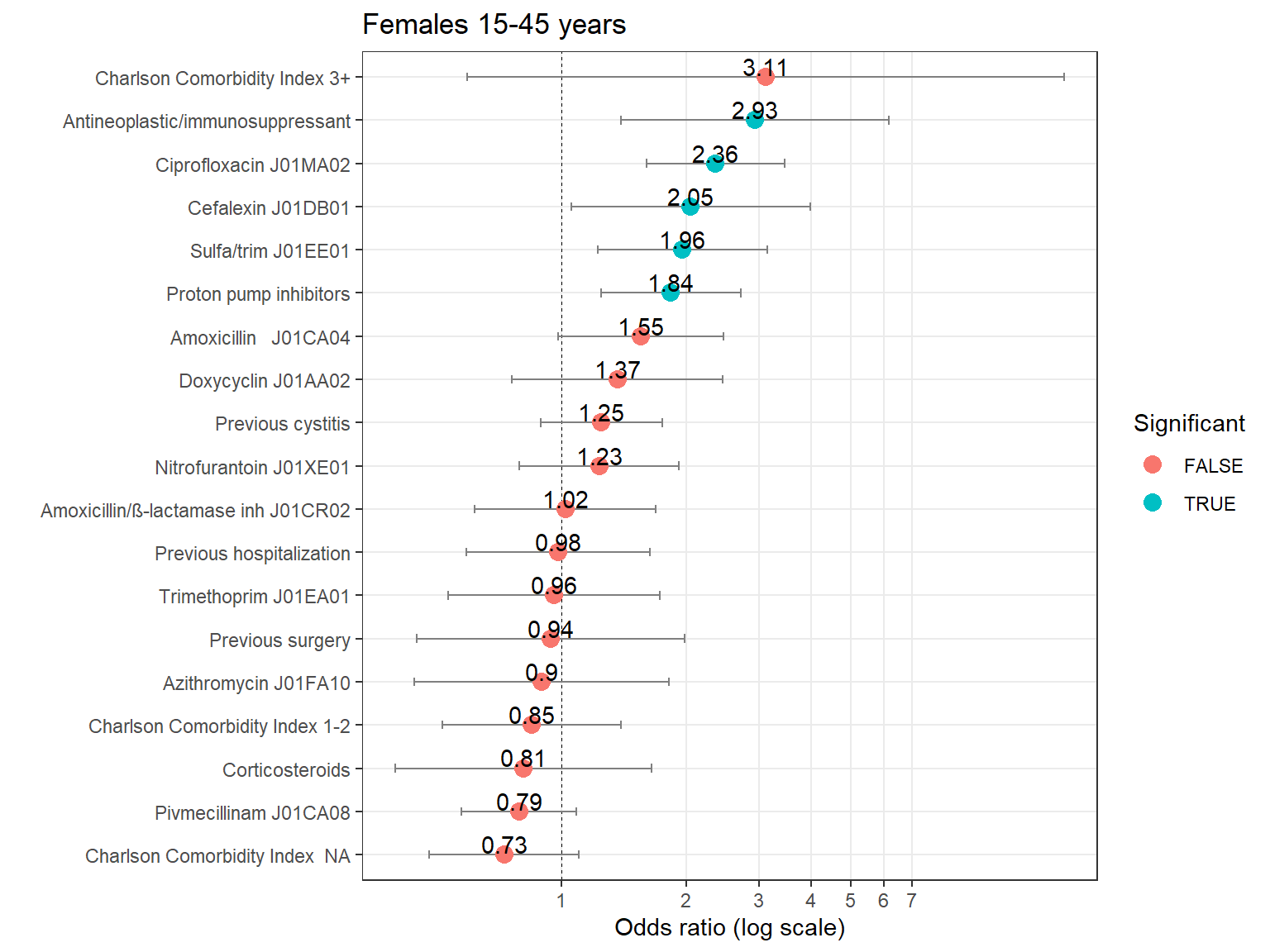


1. **Males 15-45 years (n=563)**


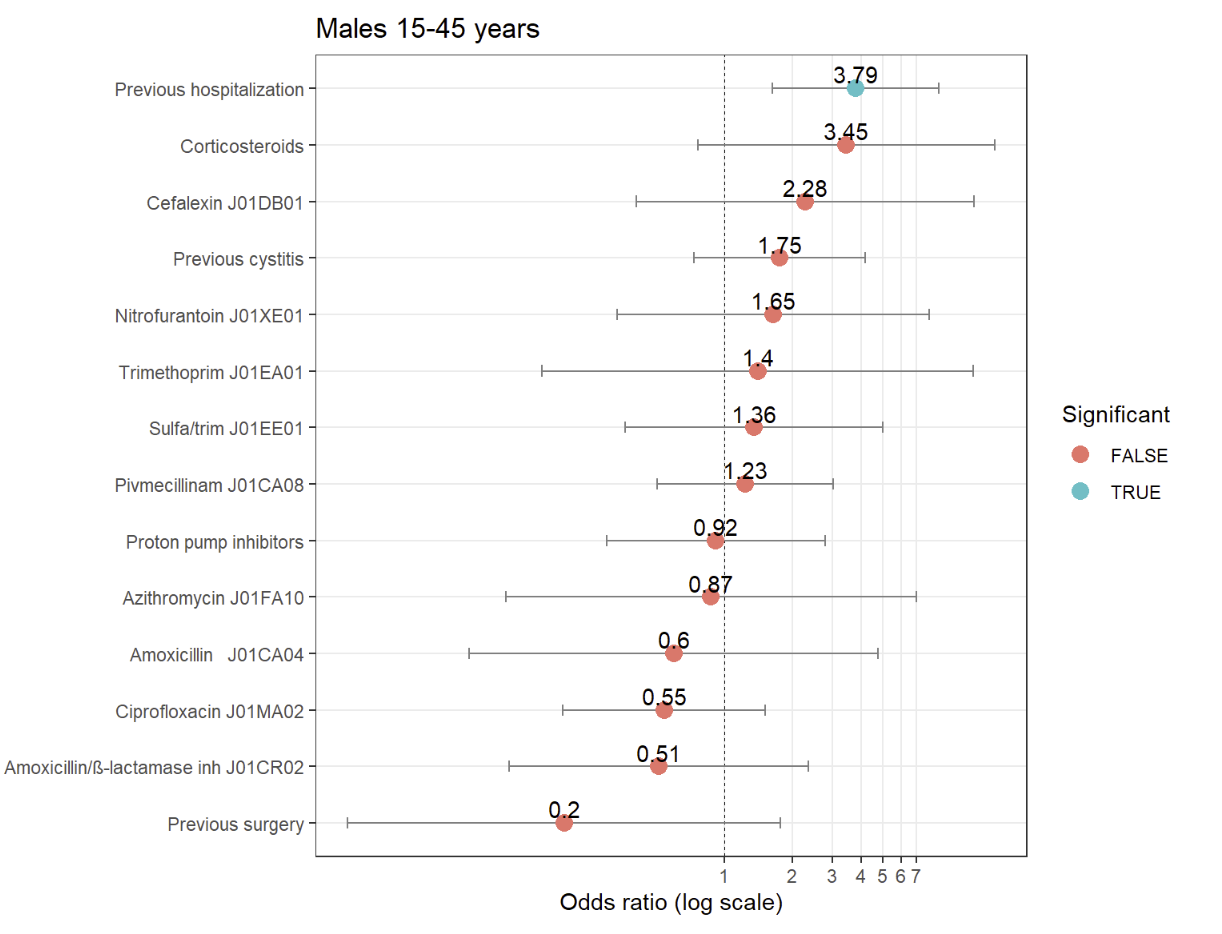


1. **Females 1-15 years (n=** **2202)**


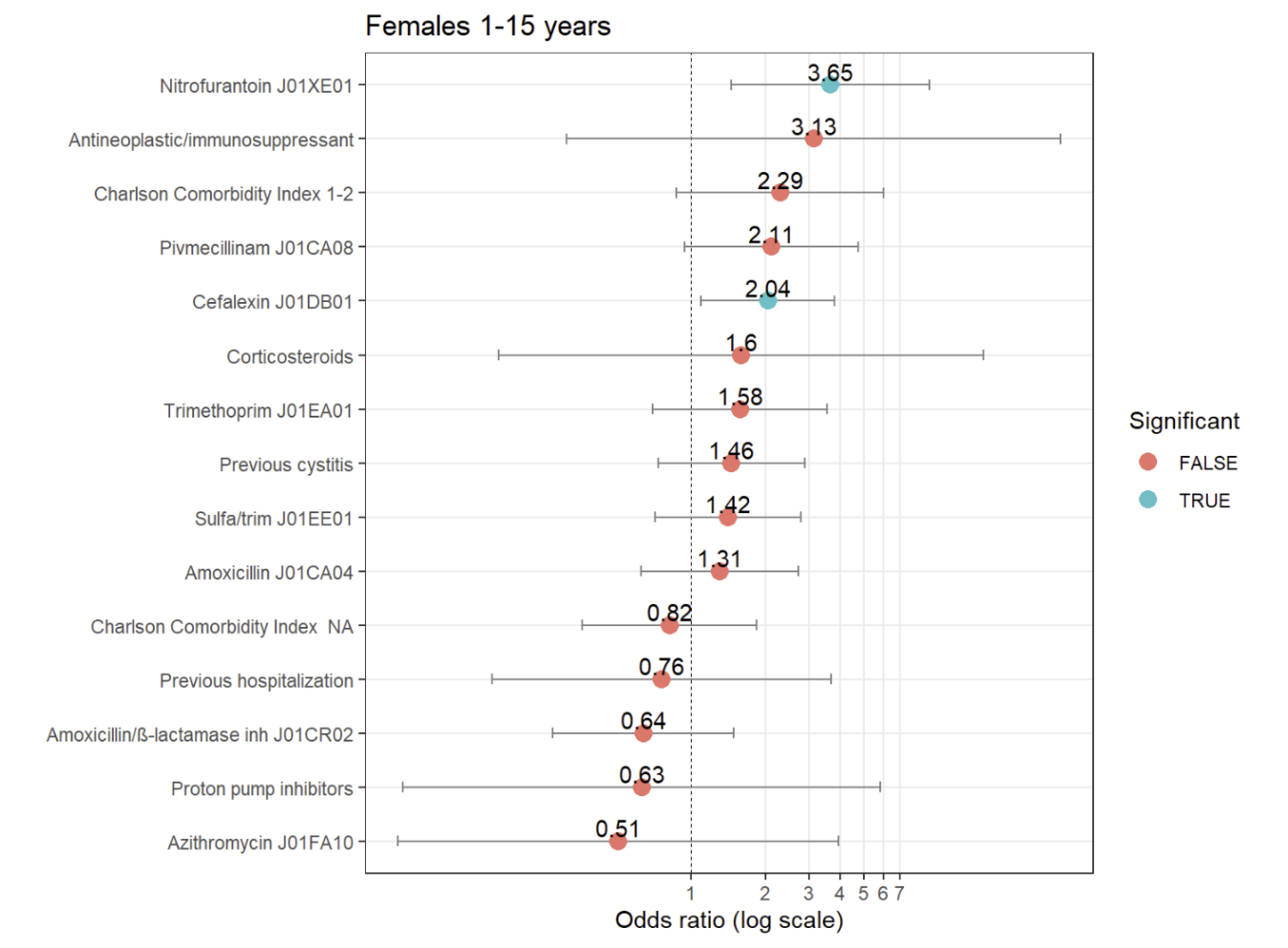


1. **Males 1-15 years (n=232)**


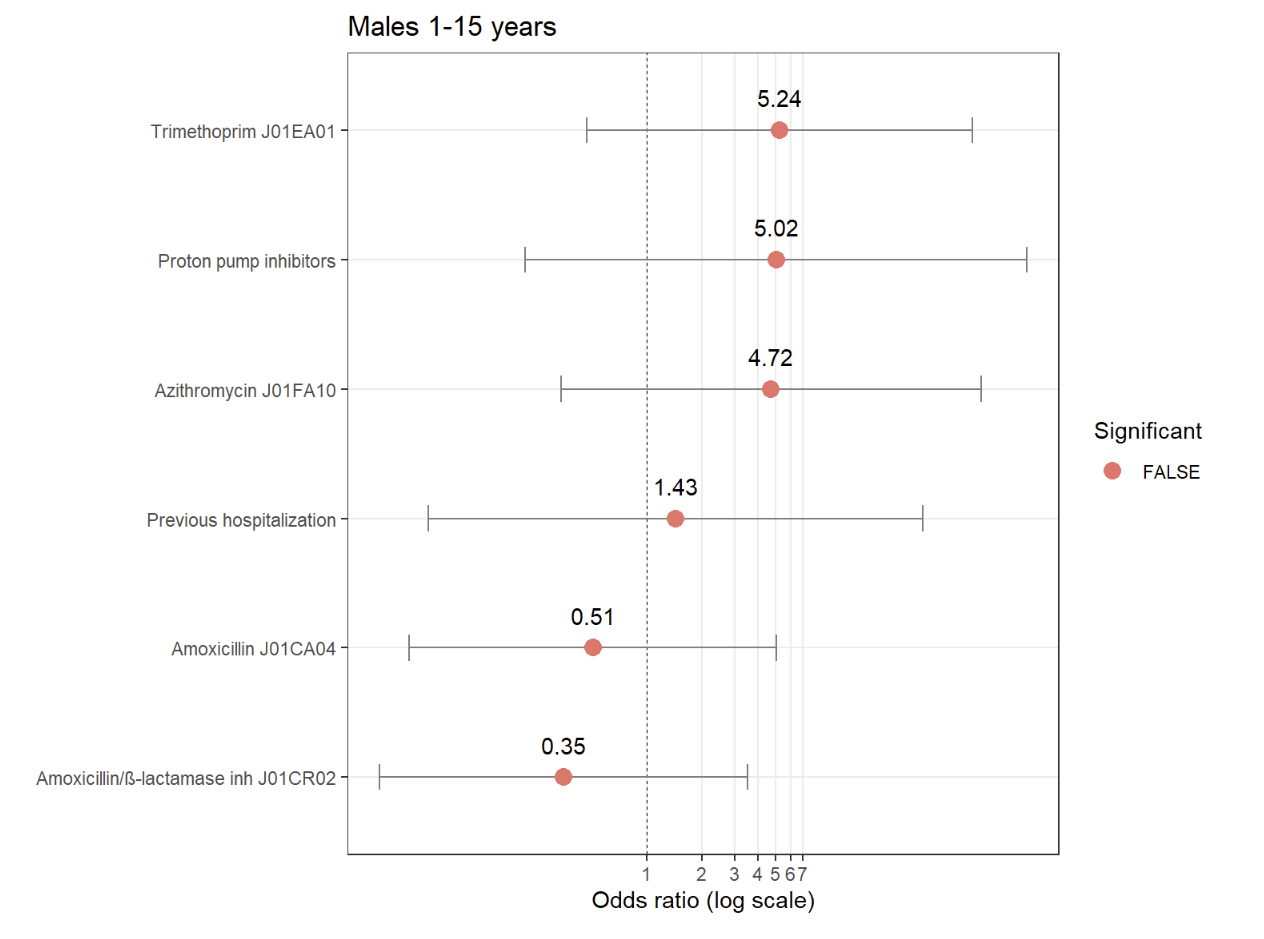

Supplement: Supplementary file 1 — Supplementary Material 1 [file 10096_2024_4882_MOESM1_ESM.docx]
